# A new baseline for fascioliasis in Venezuela: lymnaeid vectors ascertained by DNA sequencing and analysis of their relationships with human and animal infection

**DOI:** 10.1186/1756-3305-4-200

**Published:** 2011-10-14

**Authors:** M Dolores Bargues, L Carolina González, Patricio Artigas, Santiago Mas-Coma

**Affiliations:** 1 Departamento de Parasitología, Facultad de Farmacia, Universidad de Valencia, Av. Vicente Andrés Estellés s/n, 46100 Burjassot - Valencia, Spain; 2 Laboratorio de Investigaciones Parasitológicas "Dr. Jesús Moreno Rangel", Cátedra de Parasitología, Departamento de Microbiología y Parasitología, Facultad de Farmacia y Bioanálisis, Universidad de Los Andes, Urb. Campo de Oro, 5101, Mérida, Estado Mérida, Venezuela

## Abstract

**Background:**

Human and animal fascioliasis poses serious public health problems in South America. In Venezuela, livestock infection represents an important veterinary problem whereas there appear to be few human cases reported, most of which are passively detected in health centres. However, results of recent surveys suggest that the situation may be underestimated in particular areas. To obtain a baseline for future fascioliasis assessment, studies were undertaken by means of rDNA ITS-2 and ITS-1 and mtDNA *cox*1 sequencing to clarify the specific status of Venezuelan lymnaeids, their geographical distribution and fascioliasis transmission capacity, by comparison with other American countries and other continents.

**Results:**

Results obtained completely change the lymnaeid scenario known so far. The relatively rich lymnaeid fauna of Venezuela has been proven to include (i) *Lymnaea meridensis *and *L. neotropica *as the only native members, (ii) *L. cubensis *and *Pseudosuccinea columella *introduced from the Caribbean area, and (iii) *Galba truncatula *and *L. schirazensis *introduced from the Old World. The absence of representatives of the stagnicoline and *Radix *groups is remarkable. Four species are fascioliasis vectors: *G. truncatula*, *L. cubensis *and *L. neotropica*, which have the capacity to give rise to human endemic areas, and *P. columella*, which is a source of animal infection and is responsible for the spread of disease. Vector capacity in the apparently highland endemic *L. meridensis *is to be confimed, although may be expected given its phylogenetic relationships. Similarly as elsewhere, the non-transmitting *L. schirazensis *has been confused with *L. cubensis*, also with *G. truncatula *and possibly with *L. neotropica*.

**Conclusions:**

The new scenario leads to the re-opening of many disease aspects. In Venezuela, altitude appears to be the main factor influencing fascioliasis distribution. Human infection shows an altitude pattern similar to other Andean countries, although a differing highland/lowland impact on animal infection does not appear evident. The overlap of *G. truncatula*, *L. cubensis *and probably also *L. neotropica *in temperate and cold zones suggests a higher risk for human infection in mid and high altitude areas. A lymnaeid species mapping by means of DNA markers becomes a priority to determine human and animal fascioliasis distribution in Venezuela, owing to the importance of lymnaeid vectors in defining transmission and epidemiological patterns.

## Background

Fascioliasis is a pathogenic liver parasitosis caused by fasciolid flukes which affects humans and livestock species almost everywhere [1,2]. In the last two decades, this disease has emerged in many countries of Latin America, Europe, Africa and Asia [1,3]. This emergence phenomenon has partly been related to climate change [4,5], given the high dependence of both fasciolid larval stages and their freshwater lymnaeid snail vectors on climatic and environmental characteristics [6].

The infectivity of the metacercarial infective stage of isolates from different livestock species isolates have been shown to be similar [7,8], whereas the lymnaeid vector species represent a crucial factor for the epidemiology of the disease [9,10]. Geographical distribution, prevalences and intensities of both human and animal infection pronouncedly depend on the ecological characteristics (population dynamics, anthropophylic characteristics, type of water bodies, etc.) of the lymnaeid species involved in the transmission. Different lymnaeid species appear, therefore, linked to the different transmission patterns and epidemiological scenarios of this very heterogeneous disease in humans [2,11]. Thus, similarly as in other vector-borne diseases, this relationship supports the use of lymnaeids as disease biomarkers and becomes useful for mathematical modelling and remote sensing - geographical information system (RS-GIS) tools for the control of fascioliasis [12,13].

South America stands out due to the human endemic areas described in many Andean countries, including high prevalence and intensity in humans caused by *Fasciola hepatica*, such as in Chile [14], Bolivia [15-17], Peru [18,19] and Ecuador [20]. In Argentina the human fascioliasis situation seems to be underestimated [21] and in Colombia appropriate studies in risky rural areas are still pending [22].

**Figure 1:**
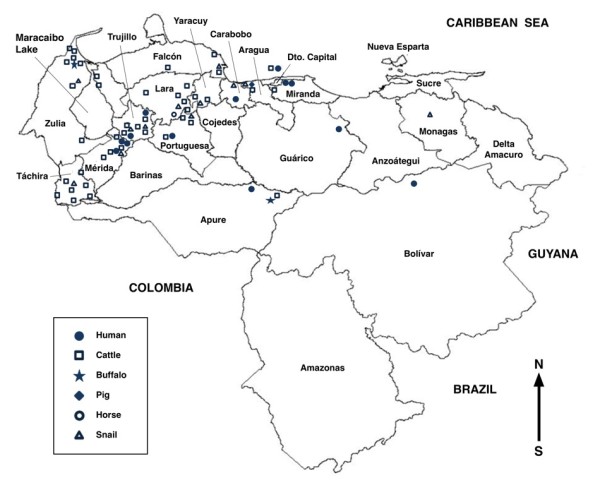
**Geographical distribution of fascioliasis in Venezuela**. Map of Venezuela showing localities where liver fluke infection in humans, livestock and lymnaeid snails has been reported.

In Venezuela, livestock infection represents an important veterinary problem in many parts of the country (Figure 1) [23]. On the contrary, human cases reported appear to be relatively few, around 50. Most of these cases have been passively detected in health centres. However, results of recent surveys give cause for concern and also indicate that the situation may be underestimated in certain areas [23]. All in all, the insufficient present fragmentary knowledge on fascioliasis suggests the need to ascertain the lymnaeid vector species present in the country, their geographical distribution, ecological characteristics and population dynamics, in order to furnish the baseline on which to design and launch the adequate studies on the disease in both humans and animals.

Only two lymnaeid species have been traditionally reported to be present in Venezuela: *Lymnaea cubensis *and *Pseudosuccinea columella *[24-27]. The first is a member of the *Galba*/*Fossaria *group, a well-known vector of fascioliasis originally described from Cuba and distributed throughout southern North America and the Caribbean [28]. *Pseudosuccinea columella *is a peculiar, widely dispersed species, believed to be originally from the region of southern North America, Central America and the Caribbean, but which has successfully colonised other continents (South America, Europe, Africa, Oceania) [29,30], and is also an efficient vector leading to increased spread of the disease [31].

This reduced list of lymnaeid species reported in Venezuela changed as the consequence of the collection of specimens of different populations of *L. cubensis *in Mérida State in March 2000. A preliminary DNA sequencing process suggested that several of these populations belonged to the morphologically very similar species *Galba truncatula*, with which it may be easily confused, similarly as it happens with other small lymnaeid species [28,32]. However, subsequent extensive DNA marker sequencing proved that indeed not only one but several different, yet similar species had been confused under the name of *L. cubensis *in Venezuela. Additionally, *Lymnaea cousini*, hithterto only known from Ecuador and Colombia [29], has recently been described and reported in Venezuela [33,34].

However, lymnaeids pose serious specimen classification difficulties when only applying malacological methods such as anatomical studies [9,10,28,35]. Moreover, intraspecific variation of shell shape is well marked in lymnaeids, although a genetic component in shell shape has been shown at least in some lymnaeid populations [36]. In the Americas, specimen classification problems are mainly related to the so-called "fossarine" or *Galba*/*Fossaria *group of numerous, very similar, usually undifferentiable lymnaeid vector species [28,32], as is the case of the aforementioned *L. cubensis*, *G. truncatula *and *L. cousini*.

The crucial implications of lymnaeid vectors for fascioliasis transmission, epidemiology and control urged the development of new tools to facilitate specimen classification, genetic characterisation of natural populations and laboratory strains, and to elucidate the systematics and taxonomy of the Lymnaeidae. This is the purpose of the worldwide lymnaeid molecular characterisation initiative [2]. Nuclear ribosomal DNA (rDNA) and mitochondrial DNA (mtDNA) markers proved useful for this endeavour in invertebrates in general, although disadvantages and limitations depending on each marker should be taken into account [37]. Their application also showed their usefulness in lymnaeids [10].

The internal transcribed spacers of the rDNA, mainly ITS-2 and secondarily ITS-1, are the most useful sequences for studies at both specific and supraspecific levels [2,10,38]. Similarly as in planorbid vectors of schistosomiasis [39], a fragment of the cytochrome c oxidase subunit I gene (*cox*1) of the mtDNA has also been used in lymnaeids [28]. However, its usefulness in lymnaeids has been recommended to be restricted to only population and close species analyses [2], due to saturation of nucleotide positions and problems posed by incomplete gene sequences [37].

The purpose of this present article is to report the results of more than ten years work by means of ITS-2, ITS-1 and *cox*1 sequencing, which are required to clarify the number of lymnaeid species present in Venezuela and to ascertain their systematic status by comparison with lymnaeids not only of neighbouring or geographically near countries but also of different continents. The final analysis offers a completely new spectrum of six lymnaeid species whose composition considerably differs from what has been noted so far. This offers a new baseline on which to design and launch appropriate studies on human and animal fascioliasis in Venezuela henceforth. The implications of this new vector scenario on the disease are finally discussed.

## Methods

### Lymnaeid snail materials

The snail specimens studied were collected in the field, from lymnaeid populations present in geographical areas with human infection and/or animal fascioliasis endemicity. Given the geographical distribution of fascioliasis concentrated in the western and north-western parts of the country (Figure 1), studies focused mainly on lymnaeid populations found in localities of selected endemic Venezuelan states and found at different altitudes, in order to (i) increase probabilities to assure finding all lymnaeid species present and (ii) sequence the lymnaeids found in areas where most human cases have been reported. No mixed populations were found. Localities furnishing the lymnaeid specimens sequenced are noted in Table 1. Coordinates and altitudes, types of habitat, month of collection, and number of specimens collected are added for each locality.

**Table 1:** Nuclear ribosomal and mitochondrial DNA haplotype code identification for lymnaeid species and populations studied from Venezuela

Lymnaeid species	Populations				Preliminary classification	rDNA ITS-2		rDNA ITS-1		mtDNA *cox*1		Combined H nomenclature
	Locality (State)	Coordinates and altitude	Habitat	No. snails (month of collection)		H	**Acc. No**.	H	**Acc. No**.	H**	**Acc. No**.	
*L. cubensis*	Magdaleno, Lago Valencia-Maracay (Aragua)	10°07'36" N 67°35'42" W 428 m	water canal Calicanto flowing into the large Lake of Valencia-Maracay	2 (November)	L. cubensis ***	4*	GenBank: F514088	B	EMBL: FN182202	b	EMBL: FN182205	L.cub-4B, *cox*1b
	Mucura, Lago Valencia-Maracay (Aragua)	10°06'38" N; 67°28'47" W 410 m	pool of stagnant water close to the large Lake of Valencia-Maracay	5 (November)	L. cubensis ***	4*	GenBank: JF514088	B	EMBL: FN182202	b	EMBL: FN182205	L.cub-4B, *cox*1b
	El Joque, Jají (Mérida)	8°35'10" N; 71°20'36" W 1995 m	irrigation canal in dairy cattle farm	40 (June)	*Lymnaea *sp. aff. cubensis ***	2	EMBL: FN182200	B	EMBL: FN182202	b	EMBL: FN182205	L.cub-2B, *cox*1b
	Estanques Lagunillas (Mérida)	°30'15" N; 71°23'41" W 1032 m	connecting canal from permanent pond inside urban area	15 (March)	*L. cubensis*	4*	GenBank: JF514088	B	EMBL: FN182202	b	EMBL: FN182205	L.cub-4B, *cox*1b

*L. neotropica*	La Linda, Güigüe (Carabobo)	10°04'53" N; 67°47'15" W 450 m	water canal at the southern part of the Lake of Valencia-Maracay	3 (May)	L. cubensis ***	2*	GenBank: JF514089	A	EMBL: AM412228	c*	GenBank: JF461485	L.neo-2A, *cox*1c
	Finca El Arenal, Tucacas (Falcón)	10°43'29" N; 68°23'25" W 30 m	irrigation canals from Aroa river in Parcelas Agrotécnicas of a farm, in the way to Las Lapas	82 (May)	L. cubensis ***	2*	GenBank: JF514089	A	EMBL: AM412228	d*	GenBank: JF461486	L.neo-2A, *cox*1d

*G. truncatula*	Iglesia Monterrey (Mérida)	8°38'39" N; 71°07'24" W 1900 m	irrigation canal along pasture grassland	47 (March)	*Galba/Fossaria *sp.	2	EMBL: AJ296271	D*	GenBank: JF514090	b*	GenBank: JF461487	G.tru-2D, *cox*1b
	San Rafael, Mucuchies A (Mérida)	8°44'42" N; 70°55'23" W 3150 m	grassland with slight slope flooded from small stream	250 (March)	*L. viatrix*	2	EMBL: AJ296271	D*	GenBank: JF514090	b*	GenBank: JF461487	G.tru-2D, *cox*1b
	San Rafael, Mucuchies B (Mérida)	8°44'46" N; 70°55'21" W 3200 m	small stream south from the village	225 (March)	*Galba/Fossaria *sp.	2	EMBL: AJ296271	D*	GenBank: JF514090	b*	GenBank: JF461487	G.tru-2D, *cox*1b
	San Isidro, Apartaderos (Mérida)	8°47'48" N; 70°51'33" W 3500 m	small stream behind the school of children San Isidro	154 (March)	*L. cubensis*	2	EMBL: AJ296271	D*	GenBank: JF514090	b*	GenBank: JF461487	G.tru-2D, *cox*1b
	Paso del Cóndor (Mérida)	8°50'41" N; 70°48'41" W 4080 m	road gutter with water descending from mountain fountain	7 (March)	*Galba/Fossaria *sp.	2	EMBL: AJ296271	D*	GenBank: JF514090	b*	GenBank: JF461487	G.tru-2D, *cox*1b

*L. schirazensis*	Hotel Valle Grande (Mérida)	8°40'28" N; 71°06'03" W 2200 m	small gutter besides external wall of hotel building	215 (March)	*Galba/Fossaria *sp.	1	GenBank: JF272601	A	GenBank: JF272603	a	GenBank: JF272607	L.schi-1A, cox1a
	Laguna Fe y Alegría (Mérida)	8°37'29" N; 70°49'33" W 1840 m	wild grass and stones besides small stream of fast running water	69 (March)	*L. cubensis*	1	GenBank: JF272601	A	GenBank: JF272603	a	GenBank: JF272607	L.schi-1A, cox1a
	La Trampa (Mérida)	8°32'25" N; 71°27'23" W 2170 m	besides small water collection on hillside	12 (May)	G. truncatula ***	1	GenBank: JF272601	A	GenBank: JF272603	a	GenBank: JF272607	L.schi-1A, cox1a

*L. meridensis*	Laguna Mucubaji (Mérida)	8°47'52" N; 70°49'32" W 3550 m	surroundings and shore of large natural pool	29 (October)	*L. cousini*	1	EMBL: FN598154	A	EMBL: FN598159	a	EMBL: FN598164	L.mer-1A, cox1a

*P. columella*	La Linda, Güigüe (Carabobo)	10°04'53" N; 67°47'15" W 450 m	water canal at the southern part of the Lake of Valencia-Maracay	2 (May)	P. columella ***	2	EMBL: FN598156	A	EMBL: FN598160	a	EMBL: FN598165	P.col-2A, cox1a
	El Valle (Mérida)	8°38'41" N; 71°07'24" W 1930 m	irrigation canal along pasture grassland close to Iglesia Monterrey	4 (March)	Lymnaeidae gen. sp.	1	EMBL: FN598155	A	EMBL: FN598160	a	EMBL: FN598165	P.col-1A, cox1a

H = haplotype; * = new haplotypes for the corresponding lymnaeid species; ** = only preliminary haplotypes due to incomplete gene sequence; *** = material collected, alcohol-fixed, preliminarily classified and furnished by colleagues

Living specimens were fixed in 70% ethanol immediately after collection in the field and stored in the same fixative until analysis.

Preliminary classification of specimens (Table 1) was, whenever possible, based on shell shape and size and morpho-anatomical characteristics traditionally considered of systematic usefulness, mainly the sexual organs. Unfortunately, sometimes snail softparts were too contracted due to fixation by alcohol 96% in cold conditions or specimens available were too small to allow a clear anatomical classification and consequently specimens were classified on shell characteristics only. In given cases, specimens were collected and preliminarily classified before 2006, when publications including phenotypic re-assessments of species were not yet available. In other cases, the preliminary classification was only at genus level, because collectors (vets, i.e., non-malacology experts) already knew that there were many species under the term of "*cubensis*" together with the great difficulties or even sometimes impossibility to diferentiate between species of *Galba*/*Fossaria*. Even in a species *a priori *easily classifiable as *P. columella *[40], its unexpected finding at very high altitude led the collectors to leave its classification open. With regard to *Galba*/*Fossaria *species, several recent articles have furnished new complete phenotypic re-descriptions that may help henceforth [22,28,41-43].

### Molecular techniques

#### DNA extraction

DNA was extracted from more than one specimen of a given population when this was deemed necessary for sequence verification. DNA was only isolated from the foot of each snail [28,44]. Snail feet fixed in 70% ethanol were used for DNA extraction procedures. After dissection under a microscope, half of the foot was suspended in 400 μl of lysis buffer (10 mM Tris-HCl, pH 8.0, 100 mM EDTA, 100 mM NaCl, 1% sodium dodecyl sulphate SDS) containing 500 μg/ml Proteinase K (Promega, Madison, WI, USA) and digested for 2 hr at 55°C with alternate shaking each 15 min. The extraction was then performed with chloroform and DNA was precipitated with isopropyl alcohol. The procedure steps were performed according to methods outlined previously [9,28,32,45]. The pellet was dried and resuspended in 30 μl sterile TE buffer (pH 8.0). This suspension was stored at -20°C until use.

#### DNA sequence amplification

Each one of the three DNA markers was PCR amplified independently for each lymnaeid specimen and each PCR product was sequenced for a bona-fide haplotype characterisation. The rDNA spacers ITS-2 and ITS-1 were amplified using primers previously described [9,28,32,46,47]. A mitochondrial DNA *cox*1 gene fragment was amplified using universal primers [48]. Amplifications were generated in a Mastercycle ep*gradient *(Eppendorf, Hamburg, Germany) using 4-6 μl of genomic DNA for each 50 μl PCR reaction. PCR conditions were 30 cycles of 30 sec at 94°C, 30 sec at 50°C and 1 min at 72°C, preceded by 30 sec at 94°C and followed by 7 min at 72°C for ITS-2 and ITS-1, and by 40 cycles of 30 sec at 90°C, 1 min at 48°C and 1 min at 72°C, preceded by 2.5 min at 94°C and followed by 10 min at 72°C for *cox*1. Ten μl of each PCR product was checked by staining with ethidium bromide on 1% Nusieve^® ^GTG agarose (FMC) gel electrophoresis, using the Molecular Weight Marker VI (Boehringer Mannheim) at 0.1 μg DNA/μl as control.

#### Purification and quantification of PCR products

Primers and nucleotides were removed from PCR products by purification on Wizard™ PCR Preps DNA Purification System (Promega, Madison, WI, USA) according to the manufacturer's protocol and resuspended in 50 μl of 10 mM TE buffer (pH 7.6). The final DNA concentration was determined by measuring the absorbance at 260 and 280 nm.

#### DNA sequencing

The sequencing of the complete rDNA ITS-2 and ITS-1 and the fragment of the mtDNA *cox*1 gene was performed on both strands by the dideoxy chain-termination method [49]. It was carried out with the Taq dye-terminator chemistry kit for ABI 3730 DNA Analyzer (Applied Biosystems, Foster City, CA, USA), using PCR primers.

#### Sequence alignments

Sequences were aligned using CLUSTAL-W version 1.8 [50] and MEGA 4.0 [51], and assembly was made with the Staden Package [52]. Subsequently, minor corrections were manually introduced for a better fit of nucleotide correspondences in microsatellite sequence regions. Homologies were performed using the BLASTN programme from the National Centre for Biotechnology information web site http://www.ncbi.nlm.nih.gov/BLAST.

#### DNA haplotype nomenclature

The codes for the sequences obtained follow the standard nomenclature proposed previously for lymnaeid snails [2,10,46]. It shall be noted that haplotype codes are only definitive in the case of complete sequences. When dealing with fragments or incomplete sequences, haplotype codes are provisional.

#### Sequence comparisons

The following sequences from GenBank-EMBL have been used for comparison analyses:

- rDNA ITS-2: *G. truncatula *H1 [EMBL: AJ296271], H2 [EMBL: AJ243017] and H3 (= *L. viatrix sensu *Ueno et al., 1975; = *L. cubensis sensu *Ueno et al., 1975) [EMBL: AJ272051] [9,28,47]; *L. cubensis *H1 [EMBL: AM412223], H2 [EMBL: FN182200] and H3 [EMBL: FN182201] [28,43], *L. neotropica *[EMBL: AM412225] [28], *L. schirazensis *H1 [GenBank: JF272601] and H2 [GenBank: JF272602] [43], *L. cousini *[EMBL: FN598153] and *L. meridensis *[EMBL: FN598154] [22]; *P. columella *H1 [EMBL: FN598155] and H2 [EMBL: FN598156] [22] and *P. columella *[GenBank: AY186751] [53].

- rDNA ITS-1: *G. truncatula *HA [EMBL: AJ243018], HB [EMBL: AJ296270] and HC (= *L. viatrix sensu *Ueno *et al*., 1975; = *L. cubensis sensu *Ueno et al., 1975) [EMBL: AJ272052] [9,28,47]; *L. cubensis *HA [EMBL: AM412226], HB [EMBL: FN182202] and HC [EMBL: FN182203] [28,43], *L. neotropica *[EMBL: AM412228] [28], *L. schirazensis *HA [GenBank: JF272603] and HB [GenBank: JF272604] [43], *L. cousini *[EMBL: FN598157] and *L. meridensis *[EMBL: FN598159] [22]; *P. columella *HA [EMBL: FN598160] [22] and *P. columella *[GenBank: AY186751] [53].

- mtDNA *cox*1 gene: *G. truncatula *from Spain [EMBL: AM494011] [28] and Germany [GenBank: EU818799] [54]; *L. cubensis cox*1a [EMBL: AM494009] and *cox*1b [EMBL: FN182205], *L. neotropica cox*1a [EMBL: AM494008] and *cox*1b [EMBL: FN356741] [28,43,55], *L. schirazensis cox*1a [GenBank: JF272607], *cox*1b [GenBank: JF272608], *cox*1c [GenBank: JF272609], and *cox*1d [GenBank: JF272610] [43], *L. cousini *[EMBL: FN598161] and *L. meridensis *[EMBL: FN598164] [22]; *P. columella *[EMBL: FN598165] [22] and *P. columella *[GenBank: AY227366] [56].

### Phylogenetic inference

Phylogenetic analysis of ITS-2 and ITS-1 combined haplotypes was performed with a Maximum Likelihood (ML) approach using PAUP version 4.0b10. ML parameters and the evolutionary model best fitting our dataset were determined using Akaike and Bayesian information criteria (AIC and BIC) [57,58], implemented in jModeltest vesion 0.1.1 [59]. Starting branch lengths were obtained using the least-squares method with ML distances.

To provide an assessment of the reliability of the nodes in the ML tree, three methods were used. First, a bootstrap analysis using 1000 replicates was made with fast-heuristic search in PAUP. Second, a distance-based phylogeny using the neighbour-joining (NJ) algorithm [60] with the ML pairwise distances was obtained and statistical support for the nodes was evaluated with 1000 bootstrap replicates, with and without removal of gapped positions. Third, a Bayesian phylogeny reconstruction procedure was applied to obtain posterior probabilities (BPP) for the nodes in the ML tree, by using the same evolutionary model as above, implemented in MrBayes 3.1 [61] with four chains during 1,000,000 generations, trees being sampled every 100 generations. The first 1000 trees sampled were discarded ("burn-in") and clade posterior probabilities (PP) were computed from the remaining trees.

Phylogenetic analyses were performed after adding the following reference sequences of rDNA ITS-2 and ITS-1 of lymnaeids stored in the databases: *L*. (*Stagnicola*) *palustris palustris *[EMBL: AJ319620, EMBL: AJ626849]; *L*. (*S*.) *fuscus *[EMBL: AJ319621, EMBL: AJ626856] [9]; *Catascopia catascopium *[GenBank: AF013143, GenBank: AF013143]; *Hinkleyia caperata *[GenBank: AF013139, GenBank: AF013139] [62]; *Radix auricularia *ITS-2 halplotype 1 [EMBL: AJ319628]; *R. balthica (= R. peregra) *ITS-2 haplotype 1 [EMBL: AJ319633] [9]. The intergenic region sequence [GenBank: AY030361] including both ITSs of the planorbid species *Biomphalaria pfeifferi *[63] was used as outgroup.

## Results

Nuclear rDNA ITS-2 and ITS-1 and mtDNA *cox*1 nucleotide sequence data reported in this paper are available in the GenBank database under the accession numbers noted in Table 1.

### *Lymnaea cubensis*

Specimens from Magdaleno and Mucura (Aragua State), Estanques Lagunillas and Jají (Mérida State), preliminarily classified as *L. cubensis *or *Lymnaea *sp. aff. *cubensis*, proved to be *L. cubensis *by ribosomal and mitochondrial DNA markers (Table 1).

#### rDNA ITS-2

**Figure 2:**
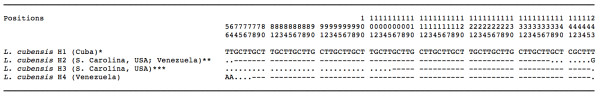
**Nucleotide variable positions and microsatellites found in the ITS-2 sequence of the *L. cubensis *populations and haplotypes studied**. Position = numbers (to be read in vertical) refer to variable positions obtained in the alignment made with MEGA 4.0. Identical = .; Indel = -. *EMBL: AM412223; ** EMBL: FN182200; *** EMBL: FN182201.

Two haplotypes were found in the populations studied. All specimens from Aragua and Estanques Lagunillas showed identical ITS-2 sequences, of 466 bp and 56.65% GC content, which is different from the three haplotypes (H1, H2, H3) available in EMBL, and is therefore here added as a new haplotype, L.cub-H4 (Table 1). The great length difference between the haplotypes L.cub-H1, H2 and H3 is related to a tetranucleotide microsatellite (CTTG) which appears in positions 51-146 of the ITS-2 alignment and which is consecutively repeated 25, 5 and 13 times in *L. cubensis *H1, H2 and H3, respectively. The new haplotype H4 is characterised by the same microsatellite repeat but appearing interruptedly (CTTG)_1_CATG(CTTG)_1_CATG(CTTG)_3_. Nucleotide and microsatellite differences in *L. cubensis *haplotypes (H1 to H4) are listed in Figure 2. The specimens from Jají population showed an ITS-2 sequence of 470 bp and 57.02% GC content, which is identical to L.cub-H2 from Sullivan Island, South Carolina, USA [EMBL: FN182200].

#### rDNA ITS-1

All the specimens from the four populations analysed present the same ITS-1 sequence, of 520 bp and a 56.35% GC content. When compared with the three ITS-1 haplotypes of *L. cubensis *available in EMBL (HA, HB, HC), this ITS-1 proved to be identical to the haplotype L.cub-HB also present in Sullivan Island, South Carolina, USA [EMBL: FN182202].

#### mtDNA cox1

Only one haplotype was detected in all specimens and populations studied, with a length of 672 bp and an AT content of 68,60%. This sequence fits exactly with the previously described L.cub-*cox*1b from South Carolina, USA [EMBL: FN182205], differing by only one mutation (T/C) with regard to L.cub-*cox*1a in position 468 of the respective alignment. In the amino acidic sequence alignment (224 aa long), both *cox*1a and *cox*1b haplotypes are identical.

### *Lymnaea neotropica*

Specimens from La Linda (Carabobo State) and Finca el Arenal, Tucacas (Falcón State), preliminarily classified as *L. cubensis*, proved to be *L. neotropica *by ribosomal and mitochondrial DNA markers (Table 1).

#### rDNA ITS-2

All the specimens showed identical ITS-2 sequence, of 415 bp and a 56.87% GC content. When compared with the ITS-2 haplotype of *L. neotropica *available in EMBL (H1), the Venezuelan sequence proved to be different, showing a shorter length due to the lack of one microsatellite repeat (AT) in positions 402-403 of the sequence alignment of both ITS-2 haplotypes. This is the first time that this haplotype has been found and has consequently been deposited in GenBank under the new code L.neo-H2 (Table 1).

#### rDNA ITS-1

All specimens studied presented the same ITS-1 sequence of 533 bp and a 56.66% GC content. This sequence was compared with the ITS-1 haplotype of *L. neotropica *available in EMBL (L.neo-HA) and proved to be identical to this haplotype also present in Peru and Argentina [EMBL: AM412228].

#### mtDNA cox1

**Figure 3:**
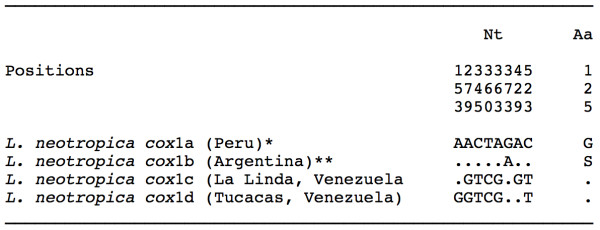
**Nucleotide and amino acid differences found in the mtDNA *cox*1 sequence of the *Lymnaea neotropica *populations studied from Venezuela**. Position = numbers (to be read in vertical) refer to variable positions obtained in the alignment made with MEGA 4.0. Nt = nucleotides; Aa = amino acids; Identical = .; Indel = -. Haplotype codes only provisional due to incomplete sequences of the gene. *EMBL: AM494008; **EMBL: FN356741.

Two haplotypes were detected in the populations studied, both identical in length (672 bp) and AT content (69,64%) but differing at two variable positions (Figure 3). When compared to the other two *cox*1 sequence haplotypes of *L. neotropica *available in EMBL (*cox*1a and *cox*1b), it proved to be different at 8 variable positions (Figure 3). Therefore, these two new haplotypes have been deposited in GenBank under the provisional codes L.neo-*cox*1c and L.neo-*cox*1d (Table 1). In the amino acidic sequence alignment (224 aa long), both *cox*1c and *cox*1d haplotypes appear to be identical. Amino acidic changes detected between the four *L. neotropica cox*1 haplotypes only concern one amino acid (G/S) in position 125 (Figure 3). Interestingly, when translated to amino acids, these Venezuelan haplotypes L.neo-*cox*1c and L.neo-*cox*1d give rise to the same amino acid sequence than the one obtained from the three *L. cubensis *haplotypes *cox*1a, *cox*1b and *cox*1c.

### *Galba truncatula*

Specimens from Iglesia Monterrey, two different populations in San Rafael de Mucuchíes, Apartaderos and Paso del Cóndor (Mérida State), preliminarily classified as *Galba/Fossaria *sp., *L. viatrix *or *L. cubensis*, proved to be *G. truncatula *by ribosomal and mitochondrial DNA markers (Table 1).

#### rDNA ITS-2

All the specimens showed an identical ITS-2 sequence, of 401 bp and a 59.10% GC content. When compared with the three ITS-2 haplotypes of *G. truncatula *available in EMBL (H1, H2, H3), this sequence proved to be identical to the previously described ITS-2 haplotype 2 (H2) for *G. truncatula *[EMBL: AJ243017].

#### rDNA ITS-1

All specimens studied showed identical ITS-1 sequence, of 504 bp and a 57.74% GC content. This sequence was compared with the three ITS-1 haplotypes of *G. truncatula *available in EMBL (HA, HB, HC) and proved to be different, with a specific mutation C instead of T in position 449 of the ITS-1 haplotype sequence alignment. This new haplotype has been deposited in GenBank under the code G.tru-HD (Table 1).

#### mtDNA cox1

**Figure 4:**
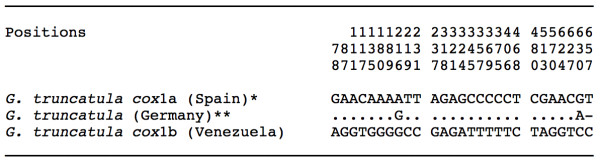
**Nucleotide differences found in the mtDNA *cox*1 gene sequence of the *Galba truncatula *populations studied from Venezuela**. Position = numbers (to be read in vertical) refer to variable positions obtained in the alignment made with MEGA 4.0. Nt = nucleotides; Identical = .; Indel = -. Haplotype codes only provisional due to incomplete sequences of the gene. *EMBL: AM494011; **GenBank: EU818799 (incomplete sequence: no haplotype available).

Only one haplotype was detected, being identical in all specimens analysed and including 672 bp and a 68.16% AT content. This haplotype proved to be different by showing 27 specific mutations when compared to the other *cox*1 sequence fragments of similar length of *G. truncatula *available in EMBL (Figure 4). The new haplotype has therefore been deposited in GenBank under the provisional code G.tru-*cox*1b (Table 1). However, both *cox*1a and *cox*1b haplotypes were identical in the 224-aa-long amino acidic sequence alignment, which means that the 27 mutations are all silent.

### *Lymnaea schirazensis*

Snail specimens collected from Hotel Valle Grande, Laguna Fe y Alegría and La Trampa (Mérida State), preliminarily classified as *Galba/Fossaria *sp., *L. cubensis *or *G. truncatula*, proved to be *L. schirazensis *after ribosomal and mitochondrial DNA marker sequencing (Table 1).

#### rDNA ITS-2

All the specimens analysed presented the same ITS-2 sequence, of 444 bp and a 53.82% GC content. When compared to the two ITS-2 haplotypes of *L. schirazensis *available in GenBank, it proved to be identical to the previously described L.schi-H1 [GenBank: JF272601]. This haplotype differs from the other haplotype known L.schi-H2 [GenBank: JF272602] in only 8 polymorphic sites, corresponding to 8 indels caused by the microsatellite repeat (TGCT), being present twice in the haplotype 1 between positions 128 and 135 of the alignment and absent in the haplotype 2.

#### rDNA ITS-1

All the lymnaeid individuals showed identical ITS-1 sequences, of 533 bp long and a 59.91% GC content. This haplotype was compared with two ITS-1 haplotypes of *L. schirazensis *available in GenBank (HA, HB) and proved to be the same as the previously described L.schi-HA [GenBank: JF272605]. Differences between this haplotype A and L.schi-HB [GenBank: JF272604] are only 1 mutation and two indels.

#### mtDNA cox1

**Figure 5:**
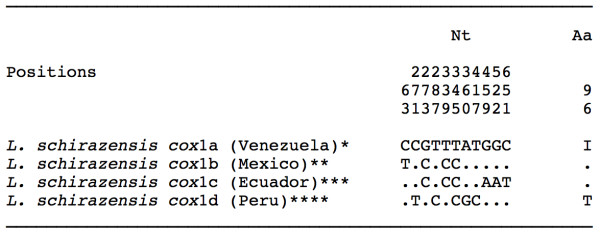
**Nucleotide and amino acid differences found in the mtDNA *cox*1 gene sequence of the *Lymnaea schirazensis *populations studied from Venezuela**. Position = numbers (to be read in vertical) refer to variable positions obtained in the alignment made with MEGA 4.0. Nt = nucleotides; Aa = amino acids; Identical = .; Indel = -. Haplotype codes only provisional due to incomplete sequences of the gene. *GenBank: JF272607, Iran, Spain, Egypt, Mexico, Peru, Dominican Republic; **GenBank: JF272608; ***GenBank: JF272609; ****GenBank: JF272610.

All of the specimens sequenced showed an identical *cox*1 nucleotide sequence, of 672 bp and with a biased AT content of 69.5%. This sequence was compared with the four *cox*1 haplotypes of *L. schirazensis *known so far and proved to be identical to the previously described haplotype L.schi-*cox*1a [GenBank: JF272607]. Nucleotide and amino acid differences between the four described haplotypes for *L. neotropica *are listed in Figure 5.

### *Lymnaea meridensis*

The specimens from Laguna Mucubaji (Mérida State), previously identified as *L. cousini*, proved by ribosomal and mitochondrial DNA marker sequences to be another species to which the name *L. meridensis *has been recently given (Table 1).

#### rDNA ITS-2

Sequence length and its slightly GC biased average nucleotide composition are 457 bp and 58.85%, respectively. This sequence corresponds to the original haplotype L.mer-H1 of the recently described species *L. meridensis *[EMBL: FN598154]. In the ITS-2 sequence alignment with the haplotype H1 of *L. cousini *[EMBL: FN598157], a high number (63) of variable positions appear (12.45%), of which 14 are mutations and 49 indels.

#### rDNA ITS-1

ITS-1 length and its slightly GC biased average nucleotide composition are 570 bp and 58.41%, respectively. This sequence corresponds to the haplotype L.mer-HA of *L. meridensis *[EMBL: FN598159]. In the ITS-1 sequence alignment of L.mer-HA with haplotypes HA and HB of *L. cousini *[EMBL: FN598157, EMBL: FN598158], a total of 47 variable positions appear (7.75%), of which 29 were mutations and 18 indels.

#### mtDNA cox1

**Figure 6:**
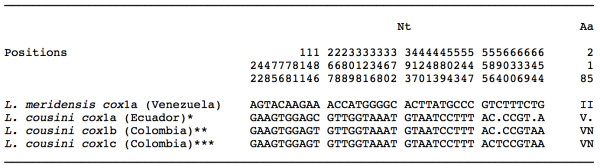
**Nucleotide and amino acid differences found in the mtDNA *cox*1 gene sequence of *Lymnaea meridensis *from Venezuela and *L. cousini *haplotypes**. Position = numbers (to be read in vertical) refer to variable positions obtained in the alignment made with MEGA 4.0. Nt = nucleotides; Aa = amino acids; Identical = .; Indel = -. Haplotype codes only provisional due to incomplete sequences of the gene. *EMBL: FN598161; **EMBL:FN598162; ***EMBL: FN598163.

This fragment has a length of 672 bp and a highly AT-biased average nucleotide composition of 69.2%, and corresponds to the haplotype *cox*1a of *L. meridensis *[EMBL: FN598164]. When compared with the three *L. cousini cox*1 provisional haplotypes available in EMBL (*cox*1a, *cox*1b and *cox*1c), the differences between both species reach 5.80%. Details on nucleotide and amino acid differences between *L. meridensis *and *L. cousini *are listed in Figure 6.

### *Pseudosuccinea columella*

The specimens from La Linda (Carabobo State) and El Valle (Mérida) previously identified as *P. columella *or ascribed to an undetermined lymnaeid (Lymnaeidae gen. sp.) respectively, proved to be *P. columella *also by ribosomal and mitochondrial DNA markers (Table 1).

#### rDNA ITS-2

Two haplotypes were found in the populations studied. All specimens from La Linda, showed identical ITS-2 sequence, of 404 bp and a biased GC content of 60.64%. This ITS-2 sequence is identical to P.col-H2 from Colombia [EMBL: FN598156]. Specimens from El Valle showed the same ITS-2 sequence of 470 bp and 57.02% GC content, which is identical to P.col-H1 from Puerto Rico [EMBL: FN598155]. Worth mentioning is the presence of T in the sequence position 6 in the La Linda population, whereas it presents C in El Valle population. When compared to the ITS-2 of *P. columella *from Cuba available in the database [GenBank: AY186751], a total of 2 mutations and 9 indels appear.

#### rDNA ITS-1

The ITS-1 sequence of both populations was the same, with a length of 536 bp and a slightly GC biased average nucleotide composition of 58.02%. This sequence was identical to that previously described for P.col-HA from Puerto Rico [EMBL: FN598160]. In a pairwise alignment comparison with *P. columella *ITS-1 from Cuba available in the database [GenBank: AY186751: 527 bp long and 58.44% GC], three indels appear in positions 262, 270 and 276. Worth noting is the presence of A in position 510, in which whether A or G were found in Cuba depending on the susceptibility or resistance characteristics of the population, respectively.

#### mtDNA cox1

Only one haplotype was detected in all specimens of the populations studied. This fragment has a length of 672 bp and a highly AT-biased average nucleotide composition of 69.2%. This sequence was compared with the other two *P. columella cox*1 sequences of similar length already published and available in databases. Nucleotide and amino acid compositions of *P. columella *from Venezuela are identical to those of the haplotype *cox*1a from Puerto Rico [EMBL: FN598165] and Australia [GenBank: AY227366].

### Phylogenetic analysis

The ML model best fitting the ITS-1 an ITS-2 combined haplotype dataset was found to be GTR+G (-Ln likelihood = 8346.73598) with a shape parameter (alpha) of 0.6622, base frequencies for A, C, G, and T of 0.20677, 0.27935, 0.25969 and 0.25419, respectively, and a proportion of invariable sites = 0. The new ITS-1 sequences obtained from the two *Radix *species *R. auricularia *and *R. balthica *used were adequately deposited in the database [EMBL: AJ319628 and EMBL: AJ319633, respectively].

**Figure 7:**
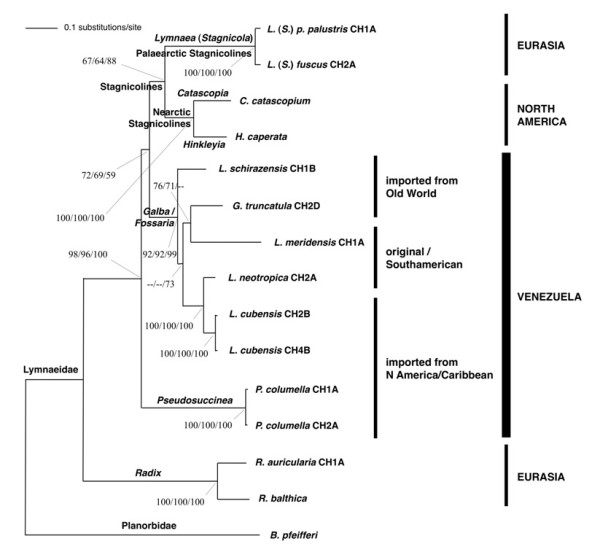
**Phylogenetic analysis of lymnaeid species from Venezuela**. Phylogenetic tree of lymnaeid species studied, obtained using the planorbid *Biomphalaria pfeifferi *as outgroup, based on maximum-likelihood (ML) estimates. Scale bar indicates the number of substitutions per sequence position. Support for nodes a/b/c: a: bootstrap with NJ reconstruction using PAUP with ML distance and 1000 replicates; b: bootstrap with ML reconstruction using PAUP with 1000 heuristic replicates; c: Bayesian posterior probability with ML model using MrBayes.

The combination of the two internal transcribed spacers in a single dataset generated a robust tree, indicating phylogenetic accordance between the two spacers in the ML tree obtained (Figure 7).

The monophyly of the ITSs haplotypes of the five lymnaeid species of the *Galba*/*Fossaria *group found in Venezuela was strongly supported (92/99/99 in NJ/ML/BBP). In this clade, *G. truncatula *does not appear clustering with other morphologically similar lymnaeids such as the New World *L. cubensis *and *L. neotropica*, nor to the phenotypically very close Old World *L. schirazensis*, but interestingly with *L. meridensis*, although supports are not high. *Lymnaea schirazensis *appears in a basal position, as a sister species, in this monophyletic group, although the clustering of the other four *Galba*/*Fossaria *species does not appear well supported.

*Pseudosuccinea columella *appears basal to the two groupings of the stagnicolines, including both Palaearctic and Nearctic species, and the *Galba*/*Fossaria *clade which comprises the *F. hepatica *main vector species. The branch of *Pseudosuccinea*, *Galba*/*Fossaria *and stagnicolines appears, moreover, well separated from the *Radix *branch, with very high supports (98/96/100 in NJ/ML/BBP).

## Discussion

### Lymnaeid species reported in Venezuela

The first report on lymnaeid snail morphology and their habitat in Venezuela, as well as their possible involvement in *F. hepatica *transmission, appeared early in the 20th century [64]. The presence and involvement of *L. cubensis *in the transmission of *F. hepatica *in different parts of the country was described from the mid-last century, mainly with regard to livestock infection [65-68], but also from places where human infection had been reported [69,70]. The presence of *P. columella *was detected for the first time in Venezuela somewhat later in a water canal of Maracay city, Aragua State [71]. Numerous multidisciplinary studies on *L. cubensis*, less numerous on *P. columella*, were performed mainly during the 1980s and first part of the 1990s, including research on distribution, biology, ecology, experimental *F. hepatica *infection, epidemiology and control in western areas [25,27,72-80].

Publications on lymnaeid snails in Venezuela have been more sporadic from the beginning of the 21st century. A high 23.3% *F. hepatica *infection prevalence in *L. cubensis *snails in endemic livestock farms of the Zulia State was noted [81]. Surprisingly high prevalences of 39-43% were also reported from non-classified lymnaeids in different habitats in another farm of Mérida [82]. After the addition of *L. cousini *[33] and inclusion of *G. truncatula*, the four aforementioned species were noted as the only lymnaeids found in Venezuela within the very recent country wide malacological review [34].

### A new lymnaeid species scenario obtained by DNA sequencing

Results obtained by DNA sequencing completely change the lymnaeid scenario in Venezuela. The new scenario of six lymnaeid species contributed in the present paper shows up to which level malacological methods may give rise to misclassifications when dealing with Lymnaeidae, mainly in problematic groups such as *Galba*/*Fossaria *[28,43]. Except *P. columella*, a species with peculiar anatomo-morphological and shell shape characteristics, which facilitate its classification mainly in the largest specimens, the remaining *L. cubensis*, *L. neotropica*, *G. truncatula*, *L. schirazensis *and *L. meridensis *may be included in *Galba*/*Fossaria *(Figure 7). This may explain why the latter four species had been overlooked under the binomium *L. cubensis *during so much time. These five *Galba*/*Fossaria *species are very similar and almost indistinguishable when young and mid-sized.

This lymnaeid fauna thus appears to be markedly rich when compared to that known in countries of the regions of Central America, the Caribbean and South America. The lymnaeid richness of Venezuela results from the overlap of (i) species of Caribbean and/or Central American origin, such as *L. cubensis *and *P. columella *[22], (ii) elements which may be considered typical or perhaps endemic of the Neotropical region, such as *L. neotropica *and *L. meridensis *[22,28], and (iii) lymnaeids indeed imported by human activities from other continents, such as *G. truncatula *of European origin and *L. schirazensis *of Asian origin [9,28,43]. This peculiar Venezuelan lymnaeid fauna is also characterised by two remarkable absences, such as the lack of representatives from the very large *Radix *and stagnicoline groups (Figure 7) [9,35]. With regard to *Radix*, Venezuela agrees with its absence in the New World, although given Old World *Radix *species were imported to the USA time ago [9]. Stagnicolines, widely spread throughout the Palaearctic region [35], are also represented by several species in the Nearctic region [62] and even by one endemic species in Mexico [83]. The absence of stagnicolines in Venezuela supports their inability to colonise warmer latitudes, which agrees with their ecological preferences for cold-mild climates throughout the Holarctic.

### Characterisation of Venezuelan lymnaeids

In *L. cubensis*, the detection of two different ITS-2 haplotypes highlights two different aspects. The fact that the combined haplotype L.cub-2B,*cox*1b of the two Aragua populations is identical to that found in South Carolina, USA, suggests a derivation from the same geographical source. Hence, the presence of this combined haplotype in lowlands as a consequence of human importation not very long ago cannot be ruled out. On the contrary, the presence of another combined haplotype differing by only two A/T transversions and a different repeat number of an interrupted microsatellite at ITS-2 level (Figure 2) suggests potential adaptive mutations to the high altitude.

The present genetic confirmation of *L. neotropica *represents the first citation of this species in Venezuela, where it could have been confused with *L. cubensis *in lowland areas. The morphological similarity between these two species is at such a level that the synonymy of *L. cubensis *and *L. viatrix *was proposed [84] largely before the molecularly-based erection of the new species *L. neotropica *for the old, northern variety B elongata of *L. viatrix *[28]. The finding of *L. neotropica *in Venezuela expands the geographical distribution of this species, hithterto only described from Peru [28] and Argentina [55], pronouncedly northward. Sequence differences, restricted to only the lack of a dinucleotide microsatellite repeat in the ITS-2 and to 6-7 mutations in *cox*1 giving rise to only one different amino acid (Figure 3), may be interpreted as peripheral populations adapted to warmer Venezuelan lowlands of a lymnaeid species apparently very widely distributed throughout South America.

The present study reports the first published DNA sequence confirmation of the presence of *G. truncatula *in Venezuela, where it has been overlooked at high altitudes but perhaps also confused with *L. cubensis *in mid altitudes around 1900-2000 m. *Galba truncatula*, of Palaearctic origin and probable man-made importation from a European source [2], has already been described in highlands of other South American countries such as Bolivia [45,47], Peru [18] and Argentina [85,86]. Whereas the detection of only one mutation in the ITS-1 may easily be assumed, the high number of 27 mutations in a 672-bp-long *cox*1 fragment when compared to other *G. truncatula *populations from elsewhere is surprising, even being silent (Figure 4).

*Lymnaea schirazensis *could have been confused in Venezuela with *L. cubensis *over a long time period and also with *G. truncatula *since the first detection of the latter in Mérida State in March 2000. Such confusion, however, does not differ from that which occurred in Asia, Africa, Europe, the Caribbean, Central America and South America. A recent large study has demonstrated that *L. schirazensis *and *G. truncatula *very pronouncedly differ at the level of rDNA and mtDNA, despite its marked anatomical and shell similarity: 130-139 nucleotide differences in ITS-2, 134-138 differences in ITS-1, and 57-67 mutations in *cox*1 [43]. This multidisciplinary study also allowed us to distinguish several phenotypic characteristics which help in the differentiation of both species [43]. The combined haplotype L.schi-1A, cox1a found in Venezuela is the same also detected in other countries such as Iran, Egypt, Spain and the Dominican Republic (Figure 5). This suggests a human importation way from its probable original area in the Near East of Asia whether indirectly through the Dominican Republic in the first decades of the Spanish colonisation period or perhaps directly from the Iberian Peninsula by way of commercial ship activities in subsequent periods [43].

*Lymnaea meridensis *has recently been described as a new species after the DNA sequencing characterisation of the lymnaeid population of Laguna Mucubaji, Mérida State [22], initially classified as *L. cousini *by malacological techniques [33]. Polymorphic sites are sufficiently numerous as to distinguish two species: 64 nucleotide differences (12.65% divergence) in ITS-2, 68-71 differences in ITS-1 (11.29-11.73%), and 37 differences in *cox*1 (5.5%) (Figure 6). Moreover, a detailed morphometric comparison allowed the differentiation of both species by many anatomical characteristics [22]. The finding of another lymnaeid population in the relatively near locality of Paso del Cóndor, at an altitude of 4040 m, recently ascribed to *L. cousini *[34], may most probably also concern *L. meridensis*. Both 18S gene sequence and the phylogenetic analysis based on ITS sequences supported a close evolutionary relationship between *L. meridensis *and *L. cousini*, suggesting an old common origin and a probable endemic divergence of *L. meridensis *by isolation in Venezuelan highlands [22].

Venezuelan *P. columella *show two ITS-2 haplotypes, H1 and H2, identical to those found in Puerto Rico and Colombia, respectively. The ITS-1 appears to be the same in these three countries. The *cox*1 fragment sequence is identical to that in Puerto Rico and Colombia [22] and also Autralia [56]. *Pseudosuccinea columella *is a rapidly colonising, more aquatic, more heat-tolerant species, considered to originate from Central America, the Caribbean and the southern part of North America. This lymnaeid is widely distributed throughout the world, including North, Central and South America and the Caribbean [26,87], Europe [30], Africa [88], Australia, New Zealand and even Tahiti [29,31]. Its typical presence in botanical gardens suggests its introduction with aquatic plants [88], a phenomenon especially increased in the last decades after the strong development of the trade of aquarium plants [30]. Thus, the presence of *P. columella *in Venezuela should most probably be considered the result of a human introduction in recent times, similarly as in whole South America and in the other continents.

Summing up, DNA sequencing results suggest that (i) the original lymnaeid fauna of Venezuela was indeed only composed by *L. meridensis *and perhaps also *L. neotropica*, and that (ii) *L. cubensis *and *P. columella *were introduced from the Caribbean area, and (iii) *G. truncatula *and *L. schirazensis *from the Old World through human shipping activities in recent times (Figure 7).

### Implications for human and animal fascioliasis epidemiology

Among the six lymnaeid species confirmed to be present in Venezuela, four of them are known to be good vectors of fascioliasis: *L. cubensis*, *L. neotropica*, *G. truncatula *and *P. columella*. This great diversity leads to the need of reopening several epidemiological and distributional aspects of the disease in both humans and animals.

*Galba truncatula *is considered the original and more efficient *F. hepatica *vector known [2]. Moreover, it is the only transmitter in the highest human fascioliasis hyperendemic situations known, namely in high altitude Andean areas of Bolivia [89] and Peru [18]. Such high fascioliasis transmission rates have been proved to be the consequence of life cycle modifications in both *F. hepatica *and *G. truncatula *as an adaptation response to the extreme conditions of the very high altitude [47]. In high altitude areas, fascioliasis in children is usually detected in the advanced chronic stage, which has proved to have a great morbidity impact [90-92]. This poses a question mark of concern with regard to Andean highlands in Venezuela. The recent detection of liver fluke infection in children when performing random surveys in altitude areas of Mérida State [93] suggests that the human fascioliasis situation may be underestimated, mainly in high altitude Andean areas where *G. truncatula *is present. It is well known that children do not usually attend hospitals or health centres in such rural areas, as proved by the high fascioliasis prevalences and intensities detected in schoolchildren in other Andean countries [16-18]. Besides Mérida State, *G. truncatula *has also been recently reported from several high altitude areas ranging 2032-2511 m in Táchira State [34], where fascioliasis in cattle is known from lowlands and mid altitudes [69,94] but human infection has never been reported.

*Lymnaea cubensis *and *L. neotropica *have also been found to be linked to human infection. *Lymnaea cubensis *is related to the Caribbean insular epidemiological pattern of human fascioliasis, typically represented by repeated outbreaks in a human hypoendemic area such as in Cuba [11]. In Venezuela, the presence of *L. cubensis *has been described in several areas of Trujillo State [24,73,76,78] and Portuguesa State [80], where or near to where human infection cases have been sporadically reported, as in highlands of Trujillo State such as Carache, at 1210 m [95,96] and Jajó, at 1796 m [97], although subsequent surveys did not detect human infection despite the a priori adequate characteristics of the place [98], and lowlands of Portuguesa State such as Guanare, at 183 m [99]. In the present study, *L. cubensis *has also been molecularly confirmed to be present in Mérida State up to almost 2000 m altitude, close to the 2050 m of the locality of Timotes where human infection was detected in a survey [93].

*Lymnaea neotropica *was originally described near Lima, Peru [28], where human infection has repeatedly been detected [100,101]. Peruvian *L. viatrix *(= *L. neotropica *according to [28]) has been shown to transmit fascioliasis both experimentally and in nature [102]. The transmission capacity of *L. neotropica *has also been molecularly confirmed in Argentina [55]. In Venezuela, DNA sequencing results have demonstrated the presence of *L. neotropica *in lowlands of Carabobo and Falcon States. No human case has so far been described from Falcon, but sporadic human infection has been reported from Valle del Cabriales, at 479 m [64], where animal infection is endemic [103], and Valencia city, at 430 m [104].

*Pseudosuccinea columella *plays a prominent role in *F. hepatica *transmission to animals, as in the Caribbean [105,106] and Brazil [107]. However, its aquatic ecology and habitat preferences seem to explain why this vector species has never been particularly involved in human infection. Interestingly, one mutation at the level of the ITS-1 and another at ITS-2 have proved useful in distinguishing between susceptible and resistant populations of *P. columella *in Cuba [53], although nothing evidently suggests that these mutations are linked to resistance/susceptibility. The presence of A in position 510 of the pairwise ITS-1 alignment comparison with *P. columella *from Cuba suggests that this species presents *F. hepatica*-susceptible populations in Venezuela, whereas a T in position 6 of the ITS-2 indicates that a resistance-linked mutation is also there, at least in La Linda. In Venezuela, *P. columella *has only been found isolatedly in the States of Aragua (Maracay city), Carabobo (southern part of Valencia Lake), Guárico (Corozo Pando) and Mérida (El Valle) throughout an altitude range of 63-1929 m [23,34]. This, together with the failure in refinding the populations of Maracay and Valencia in the 2006 survey [34], indicates that this exotic species has apparently not yet been able to further colonise and expand in Venezuela. This may be interpreted as the consequence of still insufficient time after a probable very recent man-made introduction. All in all, there is no evidence to support the idea that *P. columella *was the source of any of the human cases reported in Venezuelan lowlands. *Lymnaea cubensis *and the very similar *L. neotropica *were probably in the background of these sporadic infections, almost all concerning adult and old patients, passively detected in health centres in lowland areas. Indeed, *L. cubensis *has been cited in many lowlands of several States such as Aragua, Falcón, Lara, Portuguesa, Yaracuy, Zulia, Barinas, Trujillo, Sucre and Monagas [23,34].

Experimental infection assays of *L. schirazensis *have proved that fasciolid larval stages are not able to fully develop within this lymnaeid, which does, therefore, not participate in disease transmission [43]. In Venezuela, the presence of *L. schirazensis *poses a question mark on the geographical distribution of *G. truncatula *and all other *Galba*/*Fossaria *species with whose small and mid-sized specimens it may be very easily confused. One wonders whether unnoticed *L. schirazensis *specimens could be related to the different fascioliasis transmission capacities linked to different *L. cubensis *specimen size highlighted in mid-altitude localities of Trujillo State [76,77]. Efforts are needed henceforth in Venezuela to clarify the geographical distribution of each *Galba*/*Fossaria *species, in order to furnish the baseline on which to correctly analyse the epidemiological characteristics and geographical distribution of both human and animal fascioliasis.

Nothing is known about the potential capacity of *L. meridensis *to transmit *F. hepatica*. However, both very close molecular and phylogenetic relationships with the species *L. cousini *suggest that it may most probably be involved in fascioliasis transmission [22]. Studies, both in nature and in the laboratory, are needed to assess whether *L. meridensis *plays a role in fascioliasis transmission.

## Conclusions

Distribution, both in space (latitudinal, longitudinal and altitudinal) and time (seasonal, yearly), of fascioliasis markedly depends on climate factors influencing (i) presence/absence and population dynamics of the freshwater vector species and (ii) fluke development of free larval stages in freshwater and of parasitic larval stages inside the snail. Air temperature, rainfall and evapotranspiration are the climate factors that more pronouncedly affect definitive host infection incidence [5]. The only fasciolid present in Venezuela is *F. hepatica*, a fluke species well known due to its preference for temperate and cold climates [2]. This broadly fits with animal fascioliasis reports in Venezuela, which show a clear geographical trend for north-western temperate and colder zones (Figure 1) [23].

Human infection has, however, only been reported from the Capital District, Miranda, Carabobo, Portuguesa, Trujillo, Mérida and Bolívar (Figure 1) [23]. Although human reports are few when compared to other South American countries, such as Chile [14], Bolivia [15-17] and Peru [18,19], a similar altitude pattern appears. In Venezuela, human infection shows more numerous patients, more child involvement and case concentration (same locality, same school) in altitude areas of the western Andean States. Cases appear to be isolated, sporadic and affecting adult and old subjects in the eastern lowland plains. Thus, the altitudinal distribution of this lower human infection situation in Venezuela appears to be similar to that in Ecuador [20] and Argentina [21].

Hence, in Venezuela all evidence indicates that altitude is the main factor influencing fascioliasis distribution, and therefore highlights importance of accurately assessing the altitudinal distribution range of each of the lymnaeid vector species. Altitudinal data for each species noted in Table 1 furnish a first approach. The new scenario provided here implies the need to completely re-assess the distribution of each one of the six lymnaeid species in space and time with the help of the DNA marker tools. Lymnaeid mapping becomes a priority to determine the distribution of human and animal fascioliasis inside Venezuela. The overlap of *G. truncatula*, *L. cubensis *and probably also *L. neotropica *in temperate and cold zones suggests that there is a higher risk for human infection in mid and high altitude rural areas. Appropriate human surveys, mainly focusing on children, in such areas are evidently needed to verify whether overlooked fascioliasis prevalence may exist.

The overlap, in the same endemic area, of more than one lymnaeid vector species with different ecological requirements and population dynamics will unfortunately make the application of mathematical forecast indexes [12] more complicated. Increased difficulties may similarly be expected for remote sensing and geographical information system (RS-GIS) methods [13] to obtain accurate results useful for fascioliasis risk assessment and monitoring.

## Competing interests

The authors declare that they have no competing interests.

## Authors' contributions

MDB contributed to the design of the study, participated in field collections, analysed the sequences, performed the phylogenetic study, and helped to draft the manuscript. CG participated in field collections, contributed to epidemiological studies, and performed the local literature search. PA carried out the DNA sequencing processes. SMC designed and supervised the study, participated in field collections, performed the epidemiological analyses, and wrote the manuscript. All authors read and approved the final manuscript.
